# Report of a Case of Typhoid Fever Blended with Remittent Fever

**Published:** 1856-07

**Authors:** Austin W. Nichols

**Affiliations:** Buffalo, N. Y.


ART. III.—*Report of a Case of Typhoid Fever blended with Remittent Fever.* By Austin W. Nichols, M. D., Buffalo, N. Y.

Having devoted some portion of the past four years to the study of the various types of fever occurring in our country, and having perused the able article "On the Blending of Periodical and Continued Fever," in the last number of your Journal, it seemed to me a suitable time to present to your readers a case of typhoid fever, the peculiar circumstances attending which struck me very forcibly, and led me to make some observations on Remittent and Intermittent Fever in conjunction with Typhoid Fever, which I purpose laying before you as soon as time will permit.

During the summer of 1853, whilst on a visit to Dr. S., of Hanover, Dauphin Co., Pa., he invited me to see a patient of his, affected with fever. As we rode to the house, the Dr. remarked, that the prevailing diseases in that country, during the summer, were intermittent and remittent fevers, and dysentery. He had been in practice fourteen years, and had never seen in his "ride," a single case of typhoid fever, until the fall of 1850, when an *epidemic* of this fever swept through the valley, and many of his patients died.

Since that time, a few cases of typhoid had recurred every year, which he was able to diagnose very easily; but the periodical fever still continued to prevail as usual. During the epidemic of 1850, he had made several post-mortem examinations of those who had been affected with typhoid fever, and in every case the characteristic appearance of the intestines confirmed his diagnosis.

July 21st, 3, P. M. Mr. S., aged 65 years, farmer, in good circumstances, and of temperate habits, had been unwell for four or five days before taking to bed, complaining of frontal headache, and appearing careless or indifferent to objects around him; had *several chills*, *with fever succeeding*, but no perspiration; the chill and fever occurring always in the morning, but irregularly, sometimes early, sometimes late; had taken a few doses of *quinine*, which *stopped the chill and fever* for two days; at length he went to bed, because he was weak and wanted to sleep, "and thought he was bilious;" had no appetite; no cough; had vomited several times; had been harvesting a few hours each day, for some time previous to his attack. We found him, on the fourth day after taking to bed, lying on his back, sleeping; some stertor; easily aroused; somewhat deaf; had had no epistaxis; complained of his head; was slightly delirious, for the first time, last night; he muttered and talked incoherently; no subsultus; pulse 94; a slight capillary congestion; no cough; no expectoration; no pain in the chest; no appetite; considerable thirst; tongue dry and coated; no sordes; vomited a quantity of mucus and bile; no diarrhœa, but the bowels disposed to be open; no tympanites nor gurgling; no eruption; skin warm and soft; urine not preserved.

There being no necessity for active treatment, the Dr. concluded to try the effect of a mild cathartic:

℞ Hydrarg. chlorid. mit. gr. iij.,
Rhei pulv., gr. v. M. ft. pulv., j.

If it does not operate in five hours, to be followed by ol. ricini, f. ℥ss. Diet reduced to farinaceous articles. Cold water to be applied to the head.

July 22d, 4, P. M. Patient sleeping; stertorous respiration; deafness; no epistaxis; easily aroused; headache not so bad; dull and indifferent; some delirium last night; no subsultus; pulse 96; capillary congestion slight; slight cough, mucous expectoration; no pain in chest; no eruption; skin warm and soft; no appetite, thirst continues; tongue dry and coated; no sordes; no vomiting; no tympanites; no gurgling; slight tenderness on pressure over right iliac region; no dejections till the oil had been administered, at 5, P. M., yesterday; since then has had eight or nine liquid dejections; diarrhœa continues. The attendants stated that the patient had a *chill this morning, lasting fully half an hour.* Urine not preserved.

*Treatment.* Give an enema of laudanum; continue cold water to the head; take solution of quin. sulphat., 4 gr. to f. ℥j. of water, in f. ℥ss. doses, every three hours. Diet continued.

July 23d, 2, P. M. Patient somewhat better, though sleeping, and slight stertor present; more easily aroused; deafness continues; had some epistaxis; headache continues; some delirium last night; no subsultus; pulse 100; capillary congestion greater; cough and mucous expectoration slight; slight pain in chest; skin warm and moist; observed, what we thought to be, two or three of the characteristic pimples faintly marked; diarrhœa controlled; appetite, thirst, tongue, the same; no appearance of sordes; pain more marked in right iliac region; some gurgling; slight tympanitic distension; no vomiting.

At 3, P. M., a practitioner of much experience in the treatment of intermittent fever, who had been called by the friends to consult with Dr. S., arrived; after a thorough examination he pronounced the disease a "*low form*" of "*remittent fever.*"

*Treatment.* Continue the cold applications to the head; give beef-essence in addition to former diet; continue quinine in doses of gr. iss., per hour, through the night, if not sleeping.

July 24th, 8, A. M., patient some better; some nourishment has just been given to him; still indifferent to objects around him; deafness continues; no epistaxis; some headache; slightly delirious last night and early this morning; no subsultus; pulse 90; capillary congestion the same; no cough nor expectoration; no pain in the chest; skin warm and moist; slight diarrhœa; appetite better; thirst continues; tongue moist on edges, dry and coated in middle; slight appearance of sordes; no vomiting; pain evident on pressure over right iliac region, slightly over left; tympanites and gurgling present; skin of a yellow tinge; conjunctiva yellow. On looking for the eruption, we observed five or six rose-colored papules, small, pretty well developed, elevated, the redness disappearing on pressure, confined to the abdomen.

We concluded, at once, the case to be one of typhoid fever, in which there might be some biliary derangement, perhaps slight congestion of the liver.

*Treatment.* Hair to be cut close; cold water to be continued; quinia to be reduced to 2 grs. every four hours, and wine or brandy in f. ℥ss. doses, mixed with a small portion of water, every six hours; beef-essence or chicken broth, with milk porridge, very frequently.

July 25th, 8, A. M. Patient about the same; the brandy to be given every three hours, the quinia to be discontinued.

July 29th, 9, A. M. The fever progressed as typhoid fever usually does, without any point of uncommon interest, up to this date. The eruption disappeared yesterday: it was confined to the abdomen. The patient has been improving somewhat; he has, however, some nausea; the yellowness of the skin and conjunctiva continues.

July 30th, 4, P. M. This morning was *attacked with a chill, followed by slight fever, and terminating in profuse perspiration;* the *chill was moderate, the paroxysm lasting about three hours.* This is the *eighth day* since he had a chill, the *fifth since the quinia was stopped,* and the thirteenth since taking to the bed. Diarrhœa came on soon after he commenced to perspire, not severe, dejections being fœtid and yellow.

*Treatment.* In addition to the treatment and diet for the past few days, let an enema of laudanum be given, if diarrhœa should continue till 9, P. M. Let 3 grs. of quin. sulph. be given, in solution, every three hours.

July 31st, 12, M. Patient may be deemed convalescent; he is attentive to things around him; headache gone; some deafness; affected slightly by the quinia; slept well last night; pulse 80; capillary congestion gone; no cough, no expectoration, nor pain in the chest; eruption disappeared on the 28th, after being visible five days; skin and conjunctiva losing their yellow tint so marked on the 24th; no tympanites nor gurgling; very slight pain on pressure over the right iliac region, none over left; appetite returning; less thirst; tongue moist and cleaning; no sordes; urine, voided yesterday, exhibits a copious lateritious deposit, abundant in quantity, about three pints. Has had no chill, no fever, no perspiration.

The quinia was continued in diminished doses, 2 grs. every four hours; then, in a day or two, diminished to 2 grs. every six hours. On the 13th of August, the quinia was stopped; he was then regaining his strength rapidly. From this time nothing of interest recurred; the patient became as well as he was before the fever. From June till October, the periodical fever prevailed as usual; *two of Mr. S.'s family* were affected with *intermittent fever* at the *same time* that he was sick with typhoid.

In May, 1854, Dr. S. sent me a letter, in which he stated that Mr. Stoner, our patient, had imprudently exposed himself, on a warm day in *March*, and he was attacked thereafter *with a tertian ague*, which continued one week, and disappeared on the employment of quinia.

The diagnosis of this case may appear very easy; but setting aside two symptoms, the tenderness on pressure over the right iliac region, and the rose-colored pimples, what symptoms, or set of symptoms, were there present to warrant a correct diagnosis? I think, from the cases of remittent fever which I have seen, that there were none. Up to the 24th of July, many practitioners would have arrived, very possibly, at the same conclusion as did our worthy and intelligent friend who was called in, on the 23d; he had been in active practice, in a highly malarious country (or in a region where periodical fever prevailed extensively) for ten years; he was not ignorant of the symptoms attending typhoid fever. And basing his diagnosis on the previous history of the case, and the appearance of the patient before him, he came to a just conclusion as far as he was warranted from the facts which had been presented to his mind. He thought the case to be one of "remittent fever of a low form;" one which would bear stimulants, tonics, and nourishment: not a case which would justify the use of calomel nor other cathartics, nor diaphoretics, nor opiates in large doses, as in the treatment of many cases of remittent fever.

In some instances of remittent fever, and not infrequently, there may be tenderness, or a sense of uneasiness, on pressure over some portion of the abdomen; but never, as far as I am aware, over the right iliac region. Now in a district where remittent fever was a daily, almost an hourly occurrence, how easily would it be for a practitioner to overlook the value of this symptom! Especially so, if his patient presented a train of symptoms commonly called "*typhoid*."

The rose-colored papules, then, with the slight tenderness on pressure over the right iliac region, were the *only* pathognomonic symptoms in the above case.

Aside from these symptoms, the circumstances which would have justified a diagnosis of remittent fever, were these:

*First.* The prevalence of periodical fever in his family and in the neighborhood.

*Second.* His previous mode of living.

*Third.* Complaining of "feeling bilious," on taking to bed.

*Fourth.* The appearance of chills with fever occurring always in the morning, and vanishing after the exhibition of the quinia.

*Fifth.* The yellowness of the conjunctiva and skin, continuing throughout the disease.

*Sixth.* The recurrence of the chill on the fifth day after the quinia was suspended, and on the eighth day after the last chill; and the *final retreat* of the chill on the re-administration of the quinia.

*Seventh.* The *appearance* of a *tertian fever*, during the early days of spring, at a time when periodical fever was not common.

BUFFALO, June 16, 1856.

